# Nickel Nanoparticles Induce the Synthesis of a Tumor-Related Polypeptide in Human Epidermal Keratinocytes

**DOI:** 10.3390/nano10050992

**Published:** 2020-05-21

**Authors:** Javier Jiménez-Lamana, Simon Godin, Gerard Aragonès, Cinta Bladé, Joanna Szpunar, Ryszard Łobinski

**Affiliations:** 1 Universite de Pau et des Pays de l’Adour, E2S UPPA, CNRS, IPREM UMR, 5254 Pau, France; simon.godin@univ-pau.fr (S.G.); joanna.szpunar@univ-pau.fr (J.S.); Ryszard.Lobinski@univ-pau.fr (R.L.); 2 Department of Biochemistry and Biotechnology, Nutrigenomics Research Group, Universitat Rovira i Virgili, 43007 Tarragona, Spain; gerard.aragones@urv.cat

**Keywords:** nickel nanoparticles, human keratinocytes, cytotoxicity, protein induction, high resolution mass spectrometry

## Abstract

Although nickel allergy and carcinogenicity are well known, their molecular mechanisms are still uncertain, thus demanding studies at the molecular level. The nickel carcinogenicity is known to be dependent on the chemical form of nickel, since only certain nickel compounds can enter the cell. This study investigates, for the first time, the cytotoxicity, cellular uptake, and molecular targets of nickel nanoparticles (NiNPs) in human skin cells in comparison with other chemical forms of nickel. The dose-response curve that was obtained for NiNPs in the cytotoxicity assays showed a linear behavior typical of genotoxic carcinogens. The exposure of keratinocytes to NiNPs leads to the release of Ni^2+^ ions and its accumulation in the cytosol. A 6 kDa nickel-binding molecule was found to be synthesized by cells exposed to NiNPs at a dose corresponding to medium mortality. This molecule was identified to be tumor-related p63-regulated gene 1 protein.

## 1. Introduction

Nickel and its compounds have become important for many industrial applications due to their unique physical and chemical properties [1]. Nickel is widely used in modern industry for the production of nickel-containing alloys for coins, jewelry, internal surgical, dental devices, and stainless steel, or in nickel plating, as well as in the production of batteries and welding electrodes The increasing use of nickel is directly linked with increased risk of environmental and occupational exposure [2], which leads to various health problems [3].

Nickel is one of the most potent human allergens [4], being the most common cause of contact dermatitis of the skin; about 0.5–1% of males and 5–15% of females show a positive skin reaction to patch testing with nickel sulphate [5]. In addition, nickel is classified under Regulation (EC) No. 1272/2008 as carcinogenic category 2. The facility of nickel compounds to enter the cell and alter the intracellular level of nickel ions is directly related with its carcinogenic potential [6]. Although nickel-induced carcinogenesis is known to be related to molecular events [7], the molecular mechanisms of nickel carcinogenicity are still uncertain, and they require additional research, especially at the molecular level [8]. 

Nickel can exist in water-soluble, sulfidic, oxidic, metallic, and nanoparticulated form. Nickel carcinogenicity is compound-dependent since only certain forms of nickel can penetrate into the cell and alter the intracellular dose [7,9]. For instance, water-insoluble nickel compounds are understood to be much more potent carcinogens than the soluble forms [6,10]. Therefore, the identification of the molecular targets of insoluble chemical forms of nickel in human cells is critical.

Nickel nanoparticles (NiNPs) are being increasingly used in electronic applications and as catalysts and sensors. In vitro studies in mammalian cells involving NiNPs have shown evidence of the activation or up-regulation of cell signal pathways that are related to carcinogenicity, but the specific mechanisms are still not well understood [11]. The study of their uptake by cells as well as their toxicity assessment implies a case-by-case investigation due to the specific properties of metal nanoparticles [12]. The methodologies are based on the identification of the molecular-targets of Ni-compounds and of molecules to be synthetized by organisms in response to Ni exposure. The analytical approaches used to isolate, detect, and identify nickel-binding molecules were recently reviewed and discussed [13].

The goal of this work was to investigate, at the molecular level, the response of human skin cells to the exposure to NiNPs, to correlate it with their cytotoxicity and nickel uptake, and to compare the behavior of NiNPs with that of other chemical forms of nickel. For the purpose of the detection and identification of the Ni-binding forms at trace levels, advanced techniques that are based on the combination of different chromatographic techniques with the parallel elemental and molecular detection have been developed.

## 2. Materials and Methods 

### 2.1. Reagents and Chemicals

Analytical and biological reagent grade chemicals and LC-MS grade solvents were purchased from Sigma–Aldrich (St. Louis, MO, USA). Nickel nanoparticles (NiNPs, ≥ 99% trace metal basis; information regarding synthetic pathway not provided), nickel sulfate (NiSO_4_, ReagentPlus^®^ grade), nickel chloride (NiCl_2_, ReagentPlus^®^ grade), nickel oxide (NiO, ≥ 99.995% Trace Metal Analysis), and nickel sulfide (Ni_3_S_2_, 99.7% trace metal basis) were used as representatives of different chemical forms of nickel throughout all of the study. Aqueous nickel solutions were prepared from a standard stock solution of 1000 mg L^−1^ (Sigma–Aldrich) by dilution in ultrapure water. Ultrapure water (18.2 MΩ cm) was obtained from a Milli-Q system (Millipore, Bedford, MA, USA). 

### 2.2. Cell Viability Assay

Adult human epidermal keratinocytes (HEKa) cells (Thermo Fisher Scientific, Waltham, MA, USA) were grown in EpiLife^®^ Medium (Thermo Fisher Scientific) that was supplemented with calcium in a humidified incubator with 5% CO2 at 37 °C according to the manufacturer’s instructions. The cytotoxicity of nickel nanoparticles and other chemical forms of nickel to the keratinocytes cells was quantitatively measured using the colorimetric 3-(4,5-dimethylthiazol-2-yl)-2,5-diphenyltetrazolium bromide (MTT) reduction assay [14], according to the International Standard Methods for Biological Evaluation of Medical Devices [15]. 

A suspension of HEKa cells was placed in a 96-well plate (200 µL and 8500 cells per well). The cells were treated in the absence (control cells) or in the presence of increasing doses of NiNPs or Ni(II) (NiSO_4_, NiCl_2_, NiO, and Ni_3_S_2_) for 24 h. The concentration range of nickel used for each nickel compound is shown in Supplementary Materials (Table S1), in terms of the total amount of nickel added. Final concentrations were chosen after a first round of experiments while using a wider range. At the end of the incubation time, the medium was discarded and a new medium (200 µL) that was supplemented with 50 µL MTT (Sigma–Aldrich) solution (5 mg mL^−1^ in PBS) was added to each well. Plates were incubated at 37 °C for 4 h. After removing the medium, 200 μL DMSO (Sigma–Aldrich) was added to solubilize the formazan crystals. Finally, 25 μL of 0.1 M glycine-NaCl buffer, pH 10.5, were added and the plates were immediately read at 450 nm on an EON microplate automatic plate reader (BioTek, Winooski, VT, USA). Three independent experiments were carried out for every concentration of nickel compound tested, with six replicates each.

### 2.3. Preparation of Nickel Nanoparticles Suspension and Stability Test

A stock suspension of NiNPs was prepared in the medium before the incubation by accurately weighing a known amount of the nanopowder, followed by sonication (Branson 2510, Bransonic, Danbury, CT; nominal power and frequency: 100 W, 42 kHz ± 6%) for five minutes in order to disperse the powder and prevent the agglomeration of the nanoparticles. Longer sonication times were not used to avoid excessive heating of the suspension. The size distribution of the NiNPs stock suspension was obtained by means of Single Particle Inductively Coupled Plasma Mass Spectrometry (SP-ICPMS) (Figure S1), showing a median diameter of 46 nm and no agglomerates. On the other hand, the stability of the NiNPs in the medium was tested. Single Particle ICPMS was utilized to obtain the nanoparticle size distribution after 24 h of contact with the medium. No agglomerates were observed, and the median diameter obtained (48 nm) was not significantly different than the median diameter that was obtained for the stock suspension, confirming that the nanoparticles were stable in the medium up to 24 h. 

### 2.4. Total Nickel Determination in Cells

The HEKa cells were seeded into T75 flasks with fresh medium to final density of 2 500 cells/cm^2^ in 10 mL for 48 h or until the cells were 70% confluent before starting the experimental treatments. The cells were treated in the absence (control cells) or presence of 10 and 200 mg L^−1^ of NiNPs, 10 and 50 mg L^−1^ of NiSO_4_, 1 and 50 mg L^−1^ of NiCl_2_, 50 mg L^−1^ of NiO, or 200 mg L^−1^ of Ni_3_S_2_ for 4 h and 24 h. After the treatment, the growth medium was removed and the cells remaining at the bottom of the flask were rinsed and homogenized with a buffer containing sucrose 0.25 M, MgCl_2_ 25 mM in Tris-Hcl 20 mM at pH 7.4. The homogenate was re-homogenized using a Vibra-CellTM ultrasonic probe (Sonics & Materials Inc., Newtown, CT) for 30 s (at a power of 30%, repeated three times) and subjected to serial centrifugation and ultracentrifugation steps in order to obtain the different subcellular fractions (Avanti J-26 XPI, Beckman Coulter, Barcelona, Spain). In a first step, the homogenate was centrifuged at 700× *g* for five min. and the pellet was resuspended in buffer and kept as the “nuclear fraction”. The supernatant was recovered and ultracentrifuged at 10,000× *g* for 10 min. and the pellet was resuspended in buffer and kept as the “mitochondrial fraction”. Finally, the supernatant was recovered and centrifuged at 20,000× *g* for 2 h. The pellet was resuspended in buffer and kept as the “microsomal fraction”, while the supernatant was recovered and kept as the “cytosolic fraction”. This subcellular fractionation procedure has been adopted from Cox and Emili [16]. 

The nickel content in culture medium, cells homogenates and subcellular fractions was determined by monitoring isotopes ^58^Ni and ^60^Ni with an Agilent 7700x ICPMS. Each sample was analysed by triplicate. For all of the experiments carried out, analyses of control blanks (i.e., medium without NiNPs or any other form of nickel added) were performed. For the total nickel determination by ICPMS, the signal that was obtained for ^58^Ni and ^60^Ni in the medium was at the level of the method blank.

### 2.5. Single Particle-ICPMS Analysis

An Agilent 7900 ICPMS (Agilent) that was fitted with platinum cones and with Single Nanoparticle Application Module for ICPMS MassHunter software (Agilent) was used for single particle analysis. The sample introduction system consisted of a concentric nebulizer and a quartz cyclonic spray chamber. ^60^Ni was monitored with a dwell time of 100 µs during a total acquisition time of 60 s, while settling time during data acquisition was eliminated. Transport efficiency was determined as 3.5% by using gold nanoparticles (AuNPs) standard reference material with a nominal diameter of 56 nm that was obtained from NIST (RM 8013, Gaithersburg, MD, USA).

### 2.6. Size Exclusion Chromatography-ICPMS

Superdex 75 10/300 GL or Superdex 200 10/300 GL columns (GE Healthcare, Pittsburgh, PA, USA) were coupled to an Agilent 7700x ICPMS (Agilent) instrument that was fitted with platinum cones. Chromatographic separations were performed by using a model 1200 series HPLC pump (Agilent) as a delivery system. The exit of the column was connected in series to an UV-visible detector (Agilent) and to the nebulizer of the ICPMS instrument that was equipped with a collision/reaction cell. Table S2 lists the operational conditions (Supplementary Materials). The Superdex 200 column was calibrated in order to assign a molecular weight to each peak obtained in the samples. Protein standards (thyroglobulin, ferritin, transferrin, superoxide dismutase, myoglobin, metallothionein, and cobalamin, purchased from Sigma–Aldrich) of known molecular weight were injected onto the size exclusion column under the same conditions as the samples. The obtained linear relationship between the logarithm of the molecular weight and the retention time was used for column calibration.

### 2.7. Hydrophilic Interaction Liquid Chromatography-ICPMS

Fractions that were collected from Size Exclusion Chromatography (SEC) column were pooled, concentrated by freeze-drying (freeze dryer Crios, Cryotec, Saint-Gély-du-Fesc, France), resuspended in ammonium acetate 100 mM pH 7.4, and injected into a SeQuant^®^ ZIC^®^-cHILIC HPLC column (3 µm, 100 Å) (Millipore) coupled to an Agilent 7900 ICPMS (Agilent) fitted with platinum cones. A 1260 Infinity Bio-Inert HPLC pump (Agilent) was used as the delivery system. The exit of the column was connected to the nebulizer of the ICPMS instrument that was equipped with a collision/reaction cell. Table S3 lists separation program and operational conditions (Supplementary Materials).

### 2.8. Hydrophilic Interaction Liquid Chromatography-ESI-FT-MS/MS 

The same Hydrophilic Interaction Liquid Chromatography (HILIC) column was directly connected to an Ultimate 3000 RSLC system (Thermo Fisher Scientific, Germering, Germany) that was coupled to an Orbitrap Fusion Lumos (Thermo Fisher Scientific, San Jose, CA, USA) high resolution mass spectrometer operated in positive mode. The latter was fitted with an Advion Triversa NanoMate ion source, operated in LC coupling and fraction collection into wells mode. Basically, the Triversa NanoMate was used to partly split the LC flow to a chip-based nanoelectrospray, while most of the flow was directed to a 96-well plate for fractions collection. Post column acidification was achieved by the addition of a 30% formic acid methanol solution (v/v) to the LC flow at a rate of 10 µL min^−1^ by the mean of a syringe pump and a tee connector followed by a mixing loop (250 mm of a 250 µm ID peek tubing). This post column acidification was applied from 25 to 45 min. over the chromatogram. At this stage, the MS acquisition method consisted in a full scan detection using a resolution of 120,000. Fractions containing a Ni compound were collected and analysed by nanoESI-MS. A full scan mass spectrum was first acquired at a resolution of 120,000 and then MS/MS scans were performed for the multiply charged precursor ion while using HCD fragmentation at different normalized collision energy (NCE) ranging from 30 to 45%. The MS/MS scan that served for fragment identification was obtained at a NCE of 40%.

### 2.9. Top-Down Protein Sequence Identification 

The acquired MS/MS spectrum was first converted from RAW to MzXML format using the software MSconvert (ProteoWizard Software Foundation). It was then deconvoluted using MS-Deconv (available online: http://bix.ucsd.edu/projects/msdeconv/) and the protein identification was then achieved while using the Top-Down proteomic software MS-Align+ (available at: http://bix.ucsd.edu/projects/msalign/msalign_manual.html) [17] and a human FASTA file obtained from Uniprot (https://www.uniprot.org). Finally, the candidate protein that was identified through the untargeted approach was evaluated through a targeted approach using the software ProSight Lite (Proteomics Center of Excellence, Northwestern University, IL, USA).

## 3. Results and Discussion

### 3.1. Evaluation of the Cytotoxicity of Nickel Nanoparticles and Other Nickel Compounds Towards Human Skin Cells

**Figure 1:**
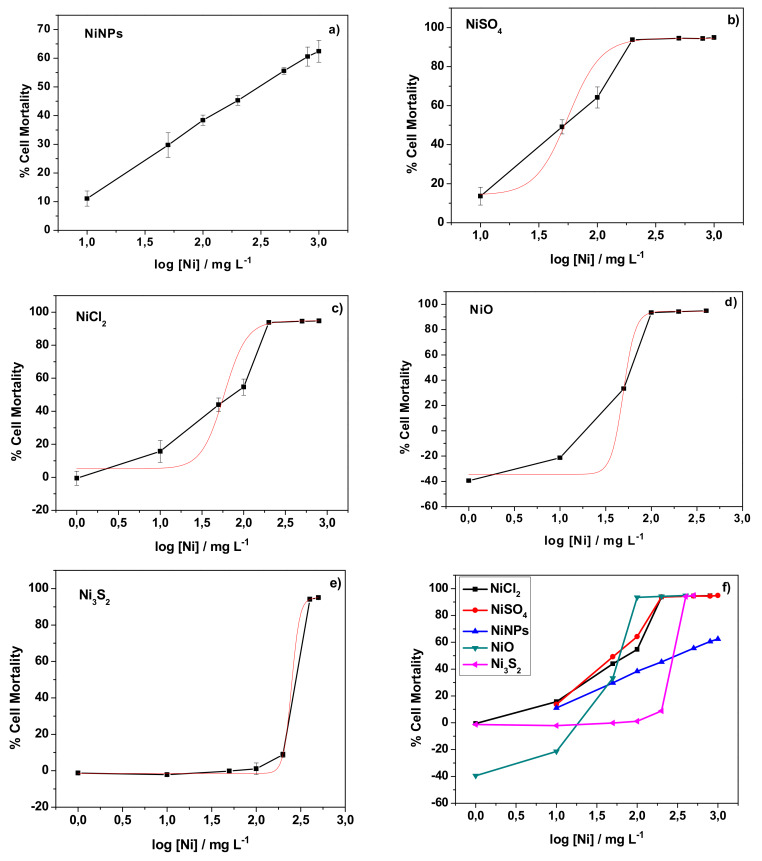
Dose-response curves obtained for the nickel compounds tested: (**a**) NiNPs; (**b**) NiSO_4_; (**c**) NiCl_2_; (**d**) NiO; and, (**e**) Ni_3_S_2_. The curves are compared in (**f**).

The viability of human skin cells exposed to NiNPs was evaluated and compared with that after exposure to other chemical forms of nickel (NiSO_4_, NiCl_2_, NiO, and Ni_3_S_2_). For this, model human skin cells were incubated with the different chemical compounds in a range of concentrations and the cytotoxicity was quantitatively measured by an MTT assay, as described in the Experimental section. Adult human epidermal keratinocytes were chosen as the cell line model. Keratinocytes are well characterized cells and they were used as cell allergy models elsewhere [18]. After the MTT assays, the dose-dependent cellular toxicity was determined for each nickel compound. Figure 1 shows the obtained dose-dependent curves.

For NiSO_4_, NiCl_2_, NiO, and Ni_3_S_2_, the dose-response curves could be fitted to sigmoid curves allowing for the determination of the LD_50_ values (Table 1). Among the compounds tested, NiO presented the highest toxicity, followed by the two soluble compounds (NiSO_4_ and NiCl_2_), which showed similar LD_50_ values. Surprisingly, the lowest toxicity was observed for nickel subsulfide. Indeed, in an experiment testing 11 Ni compounds, Ni_3_S_2_ was found to be more toxic towards AS52 cells than NiSO_4_, NiCl_2_, and NiO [19]. In any case, the results showed that even the least toxic Ni_3_S_2_ show certain toxicity towards human skin cells.

For NiNPs, the shape of the dose-response curve was different. The curve was linear, meaning that NiNPs exert a specific toxic effect towards human keratinocytes and that even low doses represent a risk. This kind of behavior, where a threshold dose is not observed, is believed to be typical of genotoxic carcinogens [20,21], although some controversy remains [22,23]. In our study, the cytotoxicity results agreed with those that were obtained for human skin cells (A431) exposed to nickel nanoparticles [24]. The genotoxic potential of metallic NiNPs was reported for human skin cells (A431) [24] and their carcinogenicity for mouse epidermis cells (JB6 cells) [25]. Note that the NiNPs used in the study with mouse epidermis cells are significantly bigger (92.3 nm) than those used in study with human skin cells and in the present one (52 and 46 nm, respectively). On the other hand, some oxide nanoparticles were reported to show a similar cytotoxicity behavior: TiO_2_ NPs in mouse fibroblast cells (L929) [26], silicon dioxide (SiO_2_) nanoparticles in human monocytes (THP-1) [27], and NiO NPs in human bronchoalveolar carcinoma-derived cells (A549) [28]. In any case, in the present study a cell mortality of around 50% was observed when the incubation was performed with a nickel concentration of 200 mg L^−1^.

### 3.2. Determination of the Nickel Uptake 

Human epidermal keratinocytes were incubated with NiNPs and the two soluble nickel salts (NiCl_2_ and NiSO_4_) for 24 h at a nickel dose corresponding to a medium cell mortality in order to study the nickel cellular uptake, as observed in the cytotoxicity study, i.e., 200 mg L^−1^ for NiNPs and 50 mg L^−1^ for the two nickel salts. After exposure, the medium was recovered, the cells were washed out with a buffer, and the total nickel content in both fractions was determined by ICPMS. For the experiments with both nickel salts, around 0.2% of the nickel mass added was found in the cells, whereas the rest of the nickel remained in the medium (Table 2). Good recoveries (>95%) were obtained for both of the experiments. 

Regarding the experiment with NiNPs, the corresponding amount of nickel found in the cells was 20 times higher. According to the obtained results, the cells took up 3.71% of the nickel added to the culture. However, the recovery obtained (Ni mass found in the medium plus Ni mass found in cells) for this experiment was very low: 8%. These results can be explained by the fact that the nanoparticles are in suspension and they may settle at the bottom of the flask during cultivation experiments. Therefore, after the treatment, they are not removed when the medium is recovered, but when the cells are rinsed and homogenized. An experiment in which NiNPs were incubated with a medium under the same conditions but without the presence of cells was performed in order to verify this hypothesis. It was found that 91% of nickel nanoparticles stuck to the bottom of the flask and the amount of nanoparticles recovered with the buffer was similar to that obtained in the experiment with cells. As this fact might bias the interpretation of the results obtained for the experiment with NiNPs, it was necessary to fractionate the cell and study the distribution of the nickel content among the different subcellular fractions in order to confirm that the nickel from NiNPs was taken up by the cells and, at the same time, to identify the target organelles of nickel.

Keratinocytes were incubated with NiCl_2_ at a dose corresponding to a medium mortality during 24 h. After the treatment, the homogenate of cells was removed from the flask and the following subcellular fractions were collected after serial centrifugation and ultracentrifugation: nuclear fraction, mitochondrial fraction, microsomal fraction, and cytosolic fraction. ICPMS determined the total nickel content in each fraction. It was found that nickel was mainly located inside the cytosol (almost 90% of the total nickel present in the initial homogenate), whereas the amount of nickel in the nucleus corresponding to 5% of the total Ni is so small that the assessment of its chemical form is below the capacity of any state-of-the art analytical technique, including this developed in this study. This result is in good agreement with other studies that were conducted with HaCaT keratinocytes that showed that nickel (in the form of NiCl_2_) was mainly accumulated in the cytosolic fraction [29,30]. Regarding NiNPs, the nickel present in the nucleus and in the mitochondria account for 10% of the intracellular nickel and the nickel amount between nucleus and mitochondria was not discriminated. The cytosol was found to be the target organelle of nickel and it was the objective of the subsequent study, according to these results. 

A significant amount of nickel (1.96 µg) was found in the cytosol of cells that were treated with NiNPs, confirming that keratinocytes were able to take up nickel when put in contact with a suspension of NiNPs and store it in the cytosol. In comparison with the results obtained for the cytosol of cells treated with soluble nickel salts (0.21 and 0.17 µg for NiCl_2_ and NiSO_4_, respectively), the amount of nickel found was four times higher, which correlates with the amount of nickel added: 1000 µg for the experiment with NiNPs vs. 250 µg for the experiments with nickel salts. However, the mass of nickel that was found in cytosols was significantly lower than the mass of nickel that was determined in the nickel homogenate (37.1 µg, Table 2), which again suggests that the majority of nickel found in the homogenate does not correspond to intracellular nickel.

**Figure 2:**
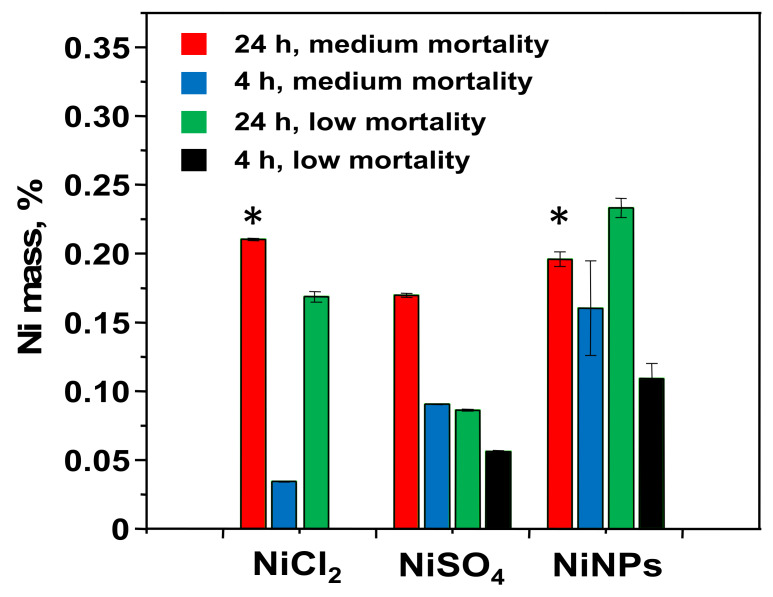
The fraction of nickel (with respect to the total nickel added to the culture) found in the cytosol of cells treated with different nickel compounds at different doses and incubation times. * Values with no significant differences among groups with a level of confidence of 95%.

In the next step, ICPMS determined the total nickel content in cytosols from cells treated with NiNPs and the other nickel compounds at two different doses (low and medium mortality) and two different incubation times (24 h and 4 h) after ultracentrifugation. For comparison purposes, results obtained results were normalized and expressed as % of nickel found in cytosol with respect to the total amount of nickel added (Figure 2). As expected, the percentage of nickel present in cytosols of cells treated with NiNPs increased when increasing the incubation time from 4 h to 24 h, a behavior that was also observed for the other nickel compounds. However, for the same incubation time, a higher percentage of nickel was found in cytosols of cells treated with the lower dose of nickel: 0.23% of nickel for a dose corresponding to low mortality against 0.20% of nickel for a dose corresponding to medium mortality. This behavior, which was not observed for the soluble nickel compounds, NiCl_2_ and NiSO_4_, suggests that the toxicity of NiNPs is not related to the amount of intracellular Ni.

The results that were obtained for NiO and Ni_3_S_2_ (Table S4) show that the highest amount of nickel in cytosols was found after the treatment with NiO, which agrees with the fact that NiO was the compound that showed the highest toxicity among all of the studied compounds (Table 1). However, no significant differences were found between the percentages of nickel found in cytosols that were treated with Ni_3_S_2_ and NiSO_4_, despite their different toxicities, which makes us conclude that the toxicity of the different nickel compounds is not solely explained by the amount of nickel present in cytosol. 

### 3.3. Physicochemical Form of Ni in Cell Cytosols 

Even though the results that were obtained in the experiments with NiNPs clearly confirmed that the cells took up a significant amount of nickel, the physicochemical form of this nickel present in the cytosol is unclear. From a toxicological and molecular point of view, it is essential to know whether nickel is able to enter the cell in the form of nanoparticles. The cytosols treated with NiNPs were analysed directly by single particle inductively coupled plasma mass spectrometry (SP-ICPMS) in order to answer this question. This technique can discriminate between the nanoparticulate and the dissolved form [31,32]. The time scan obtained only showed a steady signal characteristic of the presence of Ni in dissolved form [33] (Figure S2). However, pulses with intensities above the background, typical of the presence of nanoparticles, were not observed. If NiNPs were present in the cell, then the use of SP-ICPMS after sonication would still allow the detection of individual nanoparticles without agglomeration, as it was recently shown in literature [34,35].

Consequently, it can be concluded that all of the nickel present in cytosols comes from the oxidation/dissolution of the nanoparticles. Other metallic nanoparticles were also reported to dissolve in cellular growth media, such as silver nanoparticles (AgNPs) in Dulbecco’s Modified Eagle Medium (DMEM) [36,37,38] or in Rosewell Park Memorial Institute (RPMI) media [39]. The fact that all nickel present in cytosols was found in its dissolved form due to the dissolution of NiNPs perhaps explaining the results observed in this study. For the nickel soluble salts, all of the nickel is available for cells immediately upon addition (and hence the higher the concentration, the higher nickel available), whereas, in the case of NiNPs, the availability of nickel ions depends on the dissolution of nanoparticles. Therefore, the uptake of nickel by cells is ruled by a kinetic process and, as it was shown for other metallic nanoparticles, such as AgNPs, the ion release rate increases when the concentration of nanoparticles decreases [40]. The relative number of nickel ions released and, hence, available for the cells, was higher in the case of the experiment with a lower concentration of NiNPs (corresponding to low mortality) when compared with the experiment with a higher concentration, which explains why the percentage of nickel found was higher in the former experiment (Figure 2). On the other hand, the cytotoxicity of NiNPs might be linked to the dissolution and release of metallic ions, as it was suggested for other metallic nanoparticles, such as AgNPs [41], which may also explain the observed linear dose-response curve (Figure 1). For instance, a similar behaviour that was found for ZnO NPs was related to the cytosolic concentration of Zn^2+^ as well as to an apoptotic death pathway [42]. At this stage of research, the cause of cell death remains unknown, although apoptosis was shown to be the underlying mechanism used by ZnO NPs to induce cell death.

### 3.4. Separation of Ni-Binding Compounds

Once in the cytosol, nickel is likely to be bound to molecules already present or produced by the cells. The characterization and identification of these molecules is necessary to understand the molecular mechanisms of nickel toxicity. In this context, different analytical approaches were developed in order to separate, characterize, and identify the Ni-binding compounds present in cytosols of cells treated with NiNPs and the other nickel compounds. 

**Figure 3:**
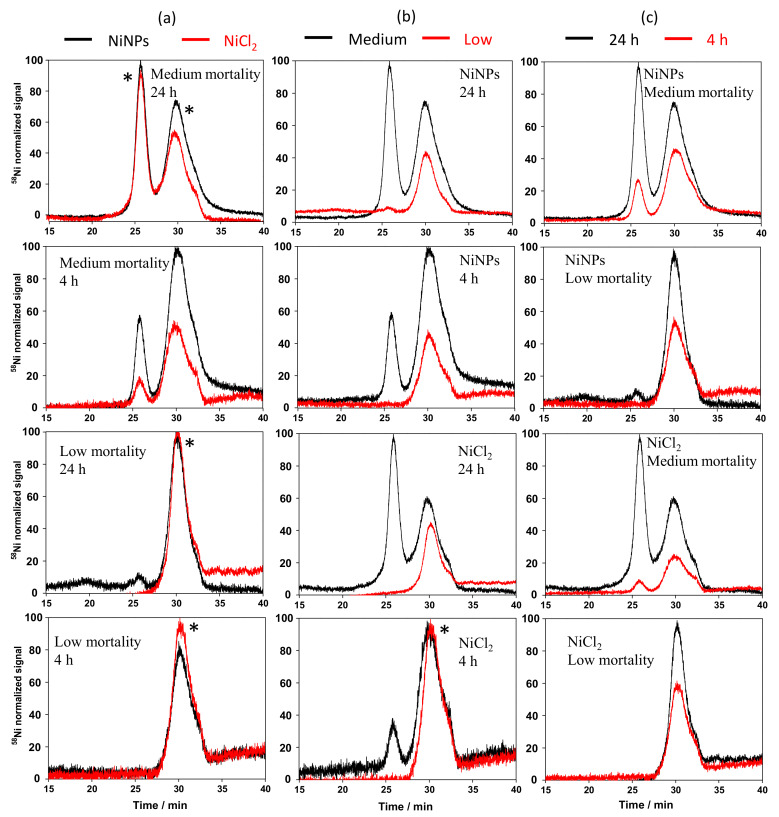
Chromatograms obtained by SEC-ICPMS for cytosols of cells treated with NiNPs and NiCl_2_ at two different nickel doses (corresponding to medium and low mortality) and two incubation times (24 and 4 h). (**a**) Comparison between treatments with NiNPs and NiCl_2_; (**b**) comparison between treatments at medium and low mortality; and, (**c**) comparison between treatments at 24 and 4 h. Asterisks indicate a pair of chromatograms (or relevant peaks) without statistical differences between them. The chromatograms were normalized for the purpose of comparison assuming the intensity of the highest peak in either chromatogram as 100%.

In the first approach, size exclusion chromatography was used for the separation of the proteins. Cytosols that were treated with NiNPs, NiCl_2_, and NiSO_4_ under the same conditions that were used in the study of the nickel uptake were injected onto a size exclusion column Superdex 200 coupled to UV-Vis and ICPMS detectors. Since similar chromatograms were obtained for the two nickel (NiCl_2_ and NiSO_4_) salts, the chromatograms obtained for NiNPs were only compared with NiCl_2_. Figure 3 shows the chromatograms obtained of cytosols of cells treated with both nickel compounds at different concentrations and incubations times. Regarding the statistical significance, the reproducibility of SEC chromatograms is +/−5% in terms of intensity and 2% in terms of elution time. The chromatograms (peaks) fitting this range were considered to be identical and are marked with an asterisk in Figure 3.

All of the chromatograms show a peak at a retention time of around 30 min. (peak II, corresponding to a compound of 1.4 kDa), whereas an additional peak eluting at around 26 min. (peak I, corresponding to a compound of 6.8 kDa) was only observed under some conditions. When comparing cytosols that were treated with both compounds, chromatograms were similar, except for the case of cells that were treated for 4 h at a dose corresponding to medium mortality, where the intensity was significantly higher for NiNPs (Figure 3a). This is in good agreement with the amount of Ni found in cytosols for the experiment with NiNPs (1.60 µg) as compared with the one that was found for the experiment with NiCl_2_ (0.09 µg). On the other hand, the differences between nickel doses were significant, as can be observed in Figure 3b. Peak I was observed for cytosols of cells that were treated with a nickel dose corresponding to medium mortality, whereas it was not observed, or it was not significant in cytosols of cells treated with a low nickel concentration (corresponding to low mortality), suggesting that this cytosolic compound is involved in the mechanisms of nickel toxicity. In addition, in the case of NiNPs, a significant increase in the intensity of peak II was observed for an increased nickel dose (1.96 and 0.12 µg of Ni found in cytosols for medium and low toxicity experiments, respectively), which was not the case in the case of cytosols that were treated with NiCl_2_. Finally, the comparison of chromatograms showed that the intensity of peaks I and II increased as a function of incubation time. This is especially significant in the case of peak I, whose intensity is higher than that of peak II for cytosols treated at a dose of medium mortality during 24 h (0.53 and 1.96 µg of Ni found in cytosols for NiCl_2_ and NiNPs experiments, respectively).

**Figure 4:**
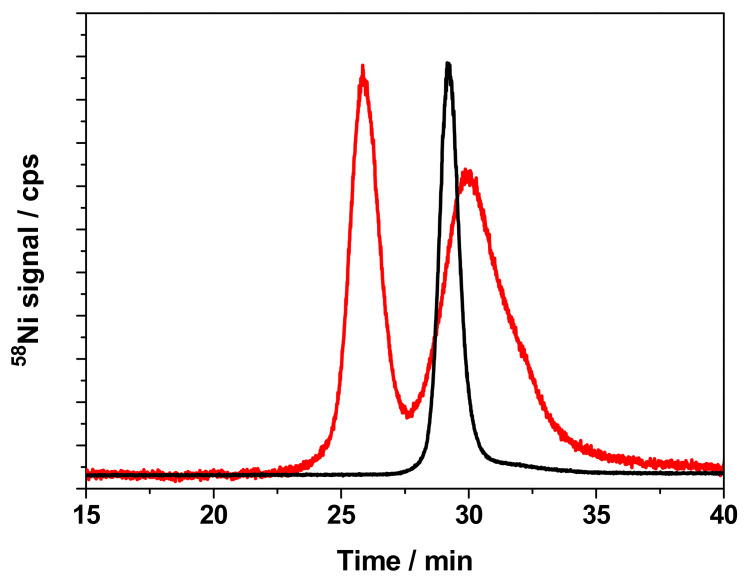
Chromatograms obtained by SEC-ICPMS for cytosols of cells treated with NiNPs at a medium mortality dose for 24 h (red line) and cytosols of untreated cells spiked with Ni^2+^ (black line).

In addition, cytosols from control cells (i.e., incubated under the same conditions, but without the presence of nickel) were spiked with Ni^2+^ and the cytosolic compounds were separated by size exclusion chromatography in the same conditions as the samples. Taking a look to the chromatogram obtained (Figure 4), peak I, which is present in the sample of cells that were treated with NiNPs at a nickel dose corresponding to medium mortality and 24 h incubation time was not observed in the cytosol of control cells spiked with Ni^2+^. This result shows that the compound that is responsible for the presence of this peak is not naturally present in cytosol, and it is only expressed by cells under stress in the presence of high doses of nickel. The peak (black line) observed in Figure 4 corresponds to a nickel-binding species that is present in the control cytosol of keratinocytes. For this reason, its identity was not investigated.

### 3.5. Identification of the Ni-Binding Compound Expressed by Keratinocytes

An analytical strategy was developed in order to identify the compound that was differentially expressed by human keratinocytes in the presence of NiNPs. The developed strategy consisted of a second chromatographic separation step coupled in parallel with ICPMS and a high-resolution electrospray mass spectrometer (ESI-FT-MS^n^). ICPMS allowed for monitoring the nickel signal of the complex, whereas ESI-FT-MS/MS provided the identity of the nickel-binding protein. 

**Figure 5:**
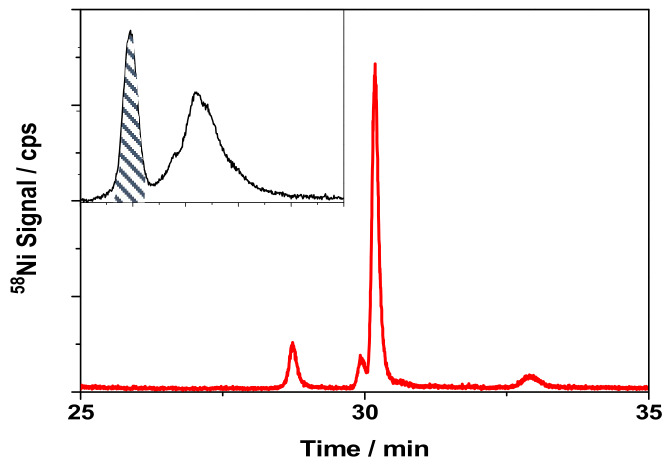
Chromatogram obtained by HILIC-ICPMS for SEC (Superdex 75, ^58^Ni detection) fraction (shown in the inset) for a cytosol of cells treated with NiNPs for 24 h at a nickel dose corresponding to medium mortality.

A better resolution of the two peaks observed is needed before performing a second dimension chromatographic step for the identification of the nickel-binding compounds corresponding to peak I. It was achieved by using a Superdex 75 column. At the same conditions of carrier composition and flow rate, a chromatogram with the peaks well resolved was obtained for cytosols of cells treated with NiNPs at a dose of medium toxicity and 24 h incubation time (Insert in Figure 5). In addition, cytosols from cells that were treated with NiO and Ni_3_S_2_ were also analyzed by SEC-ICPMS with the Superdex 75 column (data not shown). In the case of NiO, the presence of a cytosolic compound differentially expressed by the cells was observed, even after 4 h of incubation time, which was not the case of the experiments that were carried out with the other nickel compounds. The fact that NiO was found to be the nickel compound with the highest cytotoxicity might explain the expression of this protein by the cells, even at low incubation time. On the other hand, the peak I was not observed for the treatments with Ni_3_S_2_, even at a dose of medium mortality and 24 h of incubation time, which might be related with the low toxicity that was found for this compound. 

For the identification of the Ni-binding compound expressed by keratinocytes, the cytosol of a sample that was treated with NiNPs at 24 h incubation time at a dose corresponding to medium cell viability was fractionated on the Superdex 75 SEC column. The fraction corresponding to peak I was collected, concentrated, and subsequently analyzed by HILIC coupled to ICPMS. HILIC provides an efficient separation for small polar compounds, keeping the metal-biomolecule complex intact [43]. Figure 5 shows the chromatogram obtained for the SEC fraction collected from cytosols of cells treated with NiNPs; the main peak corresponds to the nickel-binding compounds of interest. 

**Figure 6:**
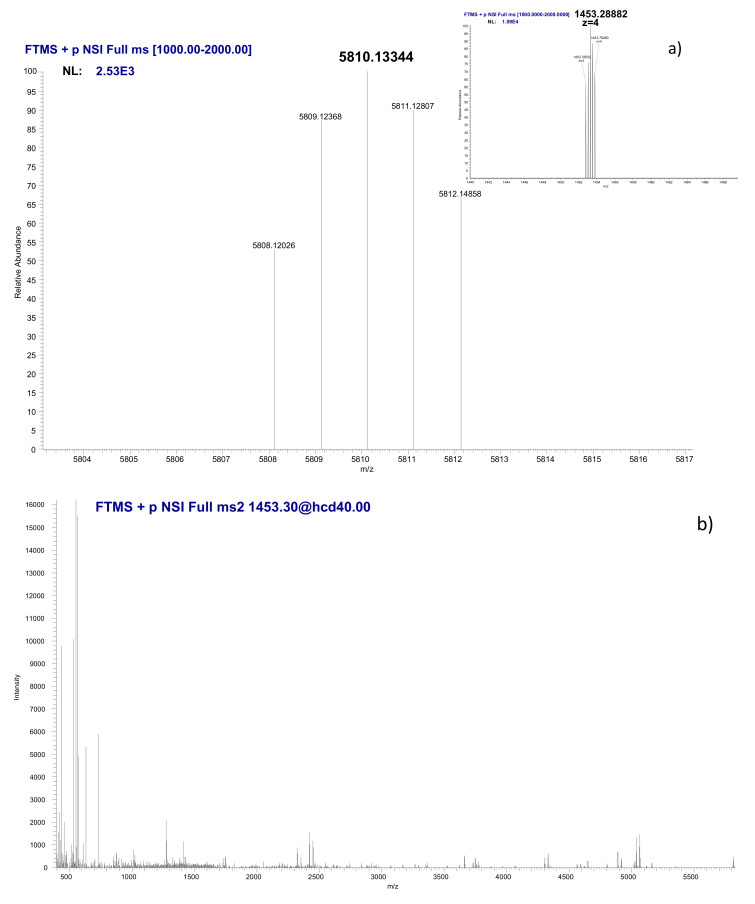

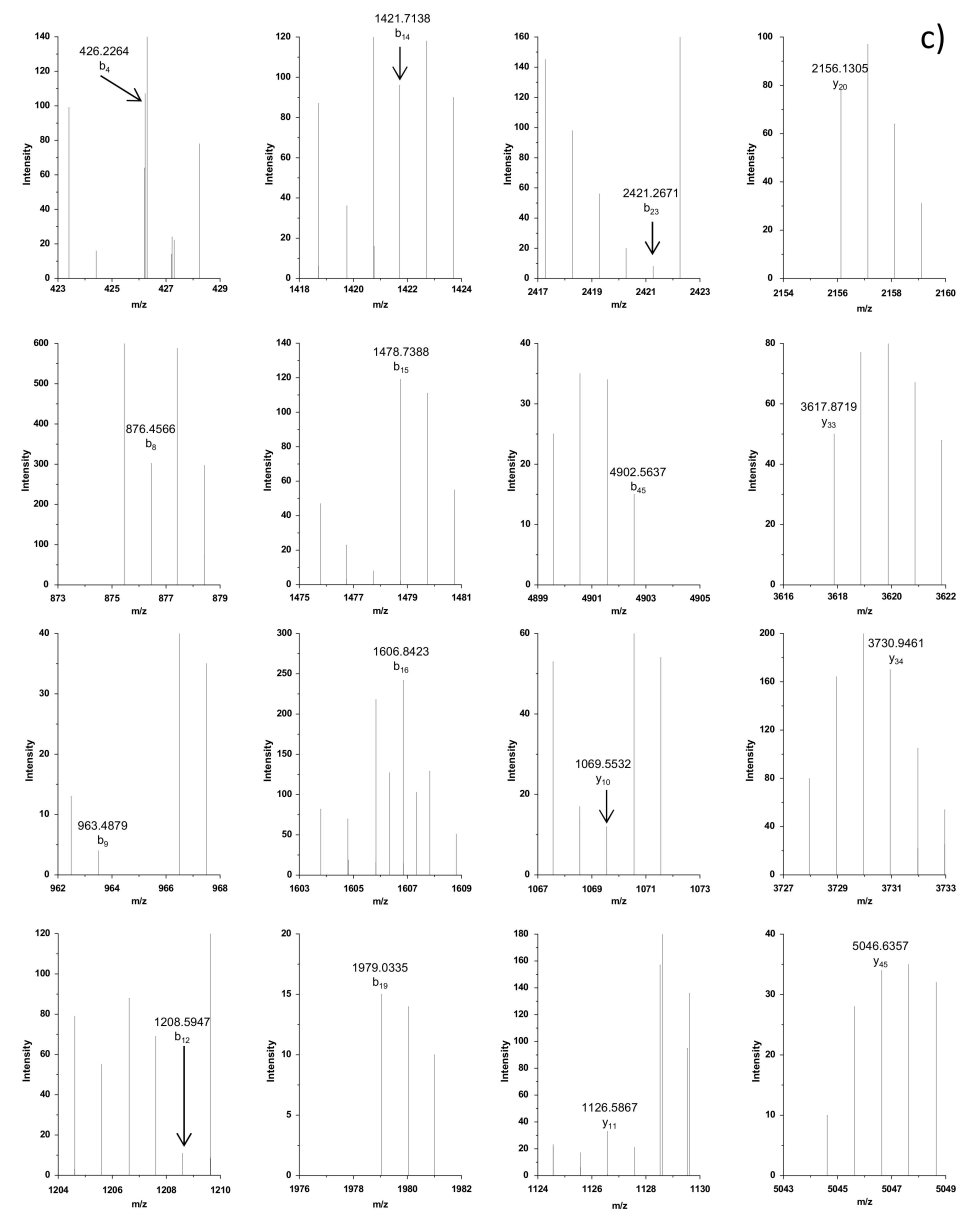
(**a**) Deconvoluted mass spectrum obtained for the SEC-HILIC Ni-containing fraction by nano-electrospray mass spectrometry (ESI-FT-MS) (infusion). The cluster of peaks at m/z of 1453.2888 Da corresponding to a quadruple charged molecule is shown as insert (**b**) MS^2^ fragmentation spectrum obtained by high-energy collisional dissociation (HCD) for the *m/z* 1453. (**c**) zooms on the 16 peak ions having served to the identification of the sequence.

An aliquot of this SEC fraction was analyzed by HILIC coupled to ESI-FTMS under the same separation conditions. Unfortunately, no signal of a Ni-bioligand compound was obtained, which was probably due to the low protein concentration in the sample. However, the use of in chip-based electrospray ionization (NanoMate) allowed for the splitting of the flow at the exit of the column, the collection of the fraction at the retention time of the Ni-compound, and its subsequent analysis in chip-based infusion MS. Figure 6 shows the deconvoluted mass spectrum corresponding to a polypeptide of a molecular weight of 5810.13344 Da. The comparison of the molecular weight measured vs. the theoretical one (5810.0996 Da) resulted in a mass difference of 5.82 ppm. Note that a post column acidification was applied in order to remove Ni from the complex to facilitate the electrospray ionization at the retention time of the Ni-compound, as discussed elsewhere for the identification of metal-binding proteins by ESI MS [44]. Consequently, the observed molecular mass corresponds to the polypeptide ligand. Figure 6b shows the MS/MS fragmentation spectrum that was obtained by using high-energy collisional dissociation (HCD) fragmentation mode. 

**Figure 7:**
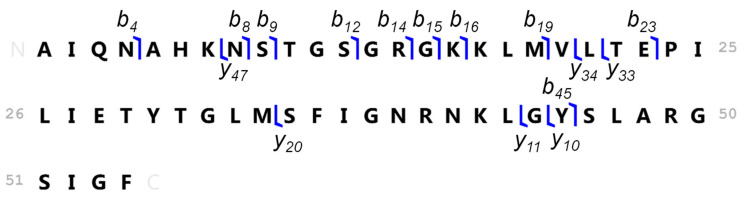
Amino acids sequence coverage of the induced nickel-binding polypeptide.

The MS and MS^2^ fragmentation data (the whole list of fragments obtained is available as Supplementary File) were processed through an untargeted Top-Down proteomics approach and different fragments were obtained (Table 3). The MS^2^ was zoomed at the vicinity of each of the fragments shown in Table 3 and, thus, 16 zooms were collected and are shown in Figure 6c. These fragments allowed for the identification of the polypeptide sequence that is shown in Figure 7. The sequence is related to a protein expressed by human epidermal cells: tumor protein p63-regulated gene 1 (TPRG-1) [45]. An open question is whether this polypeptide corresponds to the truncated isoform of the TPRG1 protein or is an enzymatic cleavage fragment. Anyhow, this question is secondary and it should not eclipse the finding that a TPRG1-related polypeptide is expressed in response to the NiNPs stress. Moreover, the truncated isoform (or its enzymatic cleavage fragment) binds strongly to nickel to let the complex pass through an HPLC column. To our knowledge, such stable polypeptide-metal complexes have not been reported from products of enzymatic cleavage, so the occurrence of a new truncated isoform of the TPRG1 protein has been assumed. Note that the binding of Ni occurs in the absence of a H2C2, which is a common Zn-finger domain that also binds Ni(II). The key to this binding is probably the presence of histidine residue and the fact that there is only one make the binding weak. Note that the binding of Ni to polypeptides containing a single histidine was reported elsewhere [46].

## 4. Conclusions

This study demonstrates, for the first time, that the dose-response curve obtained in the cytotoxicity assays for the exposure of human skin cells (keratinocytes) to NiNPs shows a linear behavior that is typical of genotoxic carcinogens, and it is different from the response to other Ni species. From the chemical point of view, at a dose corresponding to medium mortality, the exposure to NiNPs leads to the release and accumulation of Ni2+ ions in the cytosol and the biosynthesis of a 6 kDa nickel-binding molecule related to the p63-regulated gene 1 protein. It should be noted that the exposure of human cells to genotoxic carcinogen usually leads to the expression of various metabolites, but this study only focused on molecular targets of nickel. The improvement of instrumental technology providing lower detection limits will unavoidably lead to the detection of new proteins that are involved in molecular mechanisms of nickel toxicity. At the same time, future research should be oriented towards comparative proteomics or metabolomics in order to investigate the induction/suppression of ligands that are not chemically linked to Ni.

## Figures and Tables

**Table 1:** LD_50_ values obtained for the studied nickel compounds.

Compound	NiNPs	NiSO_4_	NiCl_2_	NiO	Ni_3_S_2_
LD_50_, mg L^−1^	-	56.4	58.3	49.2	249.5

**Table 2:** Total amount of nickel found in the medium and in cells treated with NiNPs and soluble nickel compounds at 24 h with a nickel dose corresponding to medium mortality.

Compound	Ni Mass Added, µg	Ni Mass in Medium, µg	Ni Mass in Cells, µg (%)	Rec, %	Ni Mass in Cytosol, µg (%)
NiNPs	1000	42.6 ± 0.1	37.1 ± 0.2 (3.71 ± 0.02)	8 ± 1	1.96 ± 0.05 (0.20 ± 0.01) *
NiCl_2_	250	248 ± 14	0.59 ± 0.01 (0.24 ± 0.01)	99 ± 5	0.53 ± 0.01 (0.21 ± 0.01) *
NiSO_4_	250	240 ± 2	0.54 ± 0.01 (0.21 ± 0.01)	96 ± 1	0.42 ± 0.01 (0.17 ± 0.01)

* Values with no significant differences among groups with a level of confidence of 95%.

**Table 3:** MS^2^ data having allowed the identification of the Ni-binding polypeptide.

Ion Type	Theoretical Mass	Observed Mass	Mass Difference (Da)	Mass Difference (ppm)
**B4**	426.2227	426.2264	0.00368	8.6
**B8**	876.4566	876.4566	−0.00002	−0.023
**B9**	963.4886	963.4879	−0.00069	−0.72
**B12**	1208.5898	1208.5947	0.00493	4.1
**B14**	1421.7124	1421.7138	0.00147	1.0
**B15**	1478.7338	1478.7388	0.00499	3.4
**B16**	1606.8288	1606.8423	0.01354	8.4
**B19**	1979.0483	1979.0335	−0.01484	−7.5
**B23**	2421.2910	2421.2671	−0.02396	−9.9
**B45**	4902.6000	4902.5637	−0.03635	−7.4
**Y10**	1069.5556	1069.5532	−0.00239	−2.2
**Y11**	1126.5771	1126.5867	0.00964	8.6
**Y20**	2156.1490	2156.1305	−0.01852	−8.6
**Y33**	3617.8915	3617.8719	−0.01961	−5.4
**Y34**	3730.9756	3730.9461	−0.02947	−7.9
**Y47**	5046.6786	5046.6357	−0.04290	−8.5

## Supplementary Materials

The following are available online at https://www.mdpi.com/2079-4991/10/5/992/s1, Supplementary File 1: Figure S1: Nanoparticle size distribution obtained by single particle-ICPMS for the stock suspension of NiNPs, Figure S2: Time scan obtained by single particle-ICPMS for cell cytosols treated with NiNPs, Table S1: Concentration range of nickel used for each nickel compound during the cytotoxicity assays, Table S2: Operational conditions of SEC-ICPMS analysis, Table S3: Operational conditions of HILIC-ICPMS analysis, Table S4: Total amount of nickel found in the medium and in cytosols treated with NiO Ni_3_S_2_ at 24 h with a nickel dose corresponding to medium mortality. Supplementary File 2: List of fragments obtained from MS^2^ fragmentation.
