# Supplementary material for: Nickel Nanoparticles Induce the Synthesis of a Tumor-Related Polypeptide in Human Epidermal Keratinocytes
Source: Nanomaterials (Basel). 2020 May 21;10(5):992. doi: 10.3390/nano10050992 (PMC7279538; doi:10.3390/nano10050992)
Supplement: Supplementary file 1 [file nanomaterials-10-00992-s001.zip › nanomaterials-797854-supplementary File 1.docx]

Supplementary Information

Nickel Nanoparticles Induce the Synthesis of a Tumor-Related Polypeptide in Human Epidermal Keratinocytes

**Javier Jiménez-Lamana ^1,^*, Simon Godin ^1^, Gerard Aragonès ^2^, Cinta Bladé ^2,†^, Joanna Szpunar ^1^, and Ryszard Łobinski ^1^**

^1^ Universite de Pau et des Pays de l’Adour, E2S UPPA, CNRS, IPREM UMR 5254, Pau, France; simon.godin@univ-pau.fr (S.G.); joanna.szpunar@univ-pau.fr (J.S.) [Ryszard.Lobinski@univ-pau.fr](mailto:Ryszard.Lobinski@univ-pau.fr) (R.L.)

^2^ Universitat Rovira i Virgili, Department of Biochemistry and Biotechnology, Nutrigenomics Research Group, 43007, Tarragona, Spain; gerard.aragones@urv.cat (G.A.); mariacinta.blade@urv.cat (C.B.)

***** Correspondence: j.jimenez-lamana@univ-pau.fr; Tel.: +33-540175037

† *Deceased*

**Table S1.** Concentration range of nickel used for each nickel compound during the cytotoxicity assays.

| Nickel compound | Total Ni concentration / mg L^−1^ | | | | | | |  |
| --- | --- | --- | --- | --- | --- | --- | --- | --- |
| NiNPs | 10 | 50 | 100 | 200 | 500 | 800 | 1000 | |
| NiSO_4_ | 10 | 50 | 100 | 200 | 500 | 800 | 1000 | |
| NiCl_2_ | 1 | 10 | 50 | 100 | 200 | 500 | 800 | |
| NiO | 1 | 10 | 50 | 100 | 200 | 400 |  | |
| Ni_3_S_2_ | 1 | 10 | 50 | 100 | 200 | 400 | 500 | |

**Table S2.** Operational conditions of SEC-ICPMS analysis.

| SEC Column | **Superdex 75 10/300 GL**  **Superdex 200 10/300 GL** |
| --- | --- |
| Carrier | Ammonium acetate 100 mM pH 7.4 |
| Flow | 0.7 mL min^−1^ |
| Injection volume | 100 µL |
| Reaction cell flow rate (H_2_) | 3.5 mL min^−1^ |
| Isotopes monitored | ^58^Ni ^60^Ni |

**Table S3.** Operational conditions of HILIC-ICPMS analysis.

| HILIC Column | **SeQuant® ZIC®-cHILIC** |
| --- | --- |
| Carrier | Ammonium formate 10 mM pH 5.5 (A)  ACN (B) |
| Flow | 0.2 mL min^−1^ |
| Injection volume | 6 µL |
| Gradient program | 0–5 min 90% B  5–45 min down to 50% B  45–50 min 50% B  50–52 min down to 35% B  52–55 min 35% B  55–60 min up to 90% B  60–70 min 90% B |
| Reaction cell flow rate (H_2_) | 5 mL min^−1^ |
| Auxiliary gas (O_2_) | 5% |
| Isotopes monitored | ^58^Ni ^60^Ni |

**Table S4.** Total amount of nickel found in the medium and in cytosols treated with NiO Ni_3_S_2_ at 24h with a nickel dose corresponding to medium mortality.

| Compound | Ni Mass Added, µg | Ni Mass in Medium, µg | Ni Mass in Cytosol, µg | Ni Mass in Cytosol, % |
| --- | --- | --- | --- | --- |
| NiO | 500 | 517 ± 2 | 1.69 ± 0.01 | 0.34 ± 0.01 |
| Ni_3_S_2_ | 2000 | 2075 ± 9 | 3.20 ± 0.07 | 0.17 ± 0.01 |

**Figure S1.** Nanoparticle size distribution obtained by single particle-ICP-MS for the stock suspension of NiNPs.

**Figure S2.** Time scan obtained by single particle-ICPMS for cell cytosols treated with NiNPs.
